# Near-infrared-emitting nanoparticles activate collagen synthesis via TGFβ signaling

**DOI:** 10.1038/s41598-020-70415-1

**Published:** 2020-08-06

**Authors:** Myung Hyun Kang, Han Young Yu, Goon-Tae Kim, Ji Eun Lim, Seunghun Jang, Tae-Sik Park, Joung Kyu Park

**Affiliations:** 1 Energy Materials Research Center, Korea Research Institute of Chemical Technology, Taejon, 34114 Korea; 2 Department of Life Sciences, Gachon University, Sungnam, 1342 Korea; 3 Center for Molecular Modeling and Simulation, Korea Research Institute of Chemical Technology, Taejon, 34114 Korea

**Keywords:** Nanoparticles, Nanoparticles

## Abstract

Research efforts towards developing near-infrared (NIR) therapeutics to activate the proliferation of human keratinocytes and collagen synthesis in the skin microenvironment have been minimal, and the subject has not been fully explored. Herein, we describe the novel synthesis Ag_2_S nanoparticles (NPs) by using a sonochemical method and reveal the effects of NIR irradiation on the enhancement of the production of collagen through NIR-emitting Ag_2_S NPs. We also synthesized Li-doped Ag_2_S NPs that exhibited significantly increased emission intensity because of their enhanced absorption ability in the UV–NIR region. Both Ag_2_S and Li-doped Ag_2_S NPs activated the proliferation of HaCaT (human keratinocyte) and HDF (human dermal fibroblast) cells with no effect on cell morphology. While Ag_2_S NPs upregulated TIMP1 by only twofold in HaCaT cells and TGF-β1 by only fourfold in HDF cells, Li-doped Ag_2_S NPs upregulated TGF-β1 by tenfold, TIMP1 by 26-fold, and COL1A1 by 18-fold in HaCaT cells and upregulated TGF-β1 by fivefold and COL1A1 by fourfold in HDF cells. Furthermore, Ag_2_S NPs activated TGF-β1 signaling by increasing the phosphorylation of Smad2 and Smad3. The degree of activation was notably higher in cells treated with Li-doped Ag_2_S NPs, mainly caused by the higher PL intensity from Li-doped Ag_2_S NPs. Ag_2_S NPs NIR activates cell proliferation and collagen synthesis in skin keratinocytes and HDF cells, which can be applied to clinical light therapy and the development of anti-wrinkle agents for cosmetics.

## Introduction

Near-infrared (NIR) irradiation has shown great potential for clinical light therapy as well as for cosmetic purposes^1–3^ and successfully been applied to photorejuvenation, photoprotection, and treatment for acne and vitiligo^4–9^ because of its long optical penetration depth of tissue. Moreover, the uniquely high NIR irradiation penetration efficiency has enabled its extensive application in therapeutic approaches to treating hypertrophic scars, including those resulting from skin aging due to skin wrinkling and skin laxity^10–18^. Clinical results have shown improvement in skin texture because NIR irradiation activates collagen synthesis and increases the amount of collagen in human dermal fibroblasts (HDF)^19,20^. Changes in collagen have been considered as a leading cause of aging and wrinkle formation because the human dermis is comprised of 90% collagen^21^. Schieke et al.^22^ reported that the skin temperature increased because the epidermal layers absorbed most of mid-IR (1.5–5.6 μm) and far-IR (5.6–10,000 μm), whereas NIR (0.8–1.5 μm) penetrated deeper to the subcutaneous tissues without causing an increase in skin temperature.

However, many studies have reported that NIR irradiation using artificial light sources may be deleterious to human skin because it raises the concentration of matrix metalloproteinase 1 (MMP-1) that damage the skin collagen. Although type I procollagen expression increases with a single NIR irradiation, it should be noted that multiple irradiations can reduce its expression^23^. Kim et al.^24^ reported that repeated NIR irradiation exposure reduced TGF-β protein expression while a single NIR irradiation increased the transforming growth factor (TGF)-β1, -β2, and -β3 expression in the human skin. TGF-β signaling activates the production of collagen and fibronectin, two important biosynthetic products in normal HDF. Therefore, in NIR-exposed human skin, any modifications in TGF-β signaling can change type I procollagen expression. Despite the benefits of NIR irradiation therapy in wounds and its potential application to the treatment of skin fibrosis, it has been reported to have some deleterious effects causing the skin temperature to increase. Research efforts towards developing NIR therapeutics to control the proliferation of human keratinocytes and collagen synthesis in the skin microenvironment have been limited, and the subject has not been fully explored. To address the challenges of the use of NIR-emitting nanoparticle (NP)-based therapeutics and to narrow the gap between current NP-based approaches and their clinical applications, there is a clear need to synthesize effective NIR-emitting NPs and to develop a promising therapeutic modality for a wide range of dermatological and cosmetic applications.

Ag_2_S NPs have been extensively studied currently as attractive NIR-emitting NPs for NIR bioimaging due to their high biocompatibility, deep tissue penetration depth, and unique absorption ability in the UV–NIR regions. These characteristics can be used for multispectral absorption applications in the UV, visible, and NIR spectral ranges because of the light absorption properties of these NPs over a broad wavelength range^25–29^. These unique optical properties, including light absorption ability within a broad wavelength range, NIR emission, and the potential application to NIR therapeutics to control the proliferation of human fibroblasts and collagen synthesis in the skin microenvironment, has motivated our research into replacing Ag_2_S NPs with an artificial NIR instrument to enhance the production of collagen. However, the development of simple methods for the preparation of high-quality and monodispersed Ag_2_S NPs is necessary for routine industrial applications, including the manufacturing of dermatological therapies and cosmetic applications.

**Figure 1:**
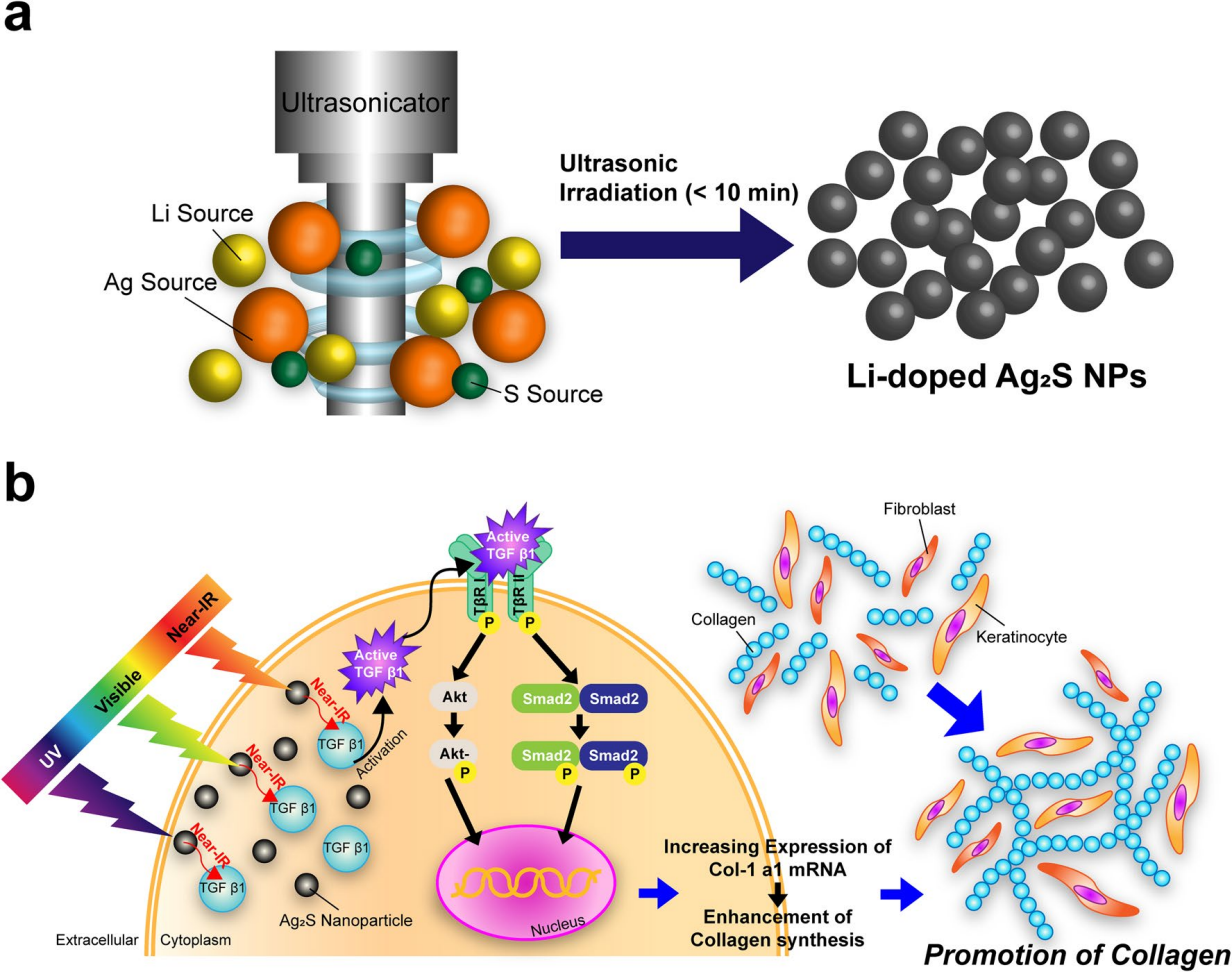
(**a**) Schematic illustration of the synthesis of Ag_2_S and Li-doped Ag_2_S NPs by using ultrasonic irradiation. (**b**) TGF-β/Smad signaling pathway activation by NIR exposure. TGF-β1 activation after irradiation with NIR causes then binding of TGF-β1 to its receptors such as tβR_p_I and tβR_p_II. This binding in turn induces the activation of the transcription factors Smad2 and Smad3, which combine with Smad4 to enter the nucleus and regulate target gene expression (type I procollagen), thereby increasing the induction of the expression of Col-1 a1 mRNA via the TGF-β/Smad signaling pathway.

Herein, we describe the simple preparation of Ag_2_S NPs and the one-pot synthesis of Li-doped Ag_2_S NPs via ultrasonic irradiation, which resulted in a dramatic enhancement of their absorption and emission capabilities in the NIR region (Fig. 1a). The effect of Li^+^ ion doping on the electronic structure of the Ag_2_S system was also investigated by using first-principles calculations, which indicated that the Li-doped Ag_2_S NPs could enhance the photoluminescence of semiconducting NPs. Finally, the effects of NIR irradiation by NIR-emitting Ag_2_S NPs on collagen production were successfully investigated.

Some wavelengths of light are absorbed by Ag_2_S NPs and Li-doped Ag_2_S NPs, which then emit NIR light to induce the activation of TGF-β1 and allows it to bind to its receptors, such as tβR_p_I and tβR_p_II. This results in the activation of the transcription factors Smad2, Smad3, and Akt, which regulate target gene expression (type I procollagen), thereby increasing the induction of the expression of COL1A1 mRNA expression via the TGF-β signaling pathway (Fig. 1b). As models in this study, we used a keratinocyte cell line and primary HDF cells.

## Results

### Preparation of NIR-emitting Ag_2_S and Li-doped Ag_2_S NPs

A simple synthesis of 10 nm Ag_2_S NPs was performed by using a sonochemical method in which the decomposition of raw materials was induced by ultrasound under ambient conditions to enhance collagen production using NIR-emitting NPs. Silver nitrate (AgNO_3_) in 1-dodecanethiol was sonicated to generate localized hot spots within the acoustic cavitation of collapsing bubbles during ultrasonic irradiation (reaction time: 10 min, power: 50%, temperature: ~ 160 ℃). Li-doped Ag_2_S NPs were synthesized by adding a suitable amount of Li^+^ to the reaction bottle, a method to fabricate undoped Ag_2_S NPs, which improves NIR emission intensity, enhancing absorption properties in the broad wavelength range to increase its general photoluminescence (PL) performance.

**Figure 2:**
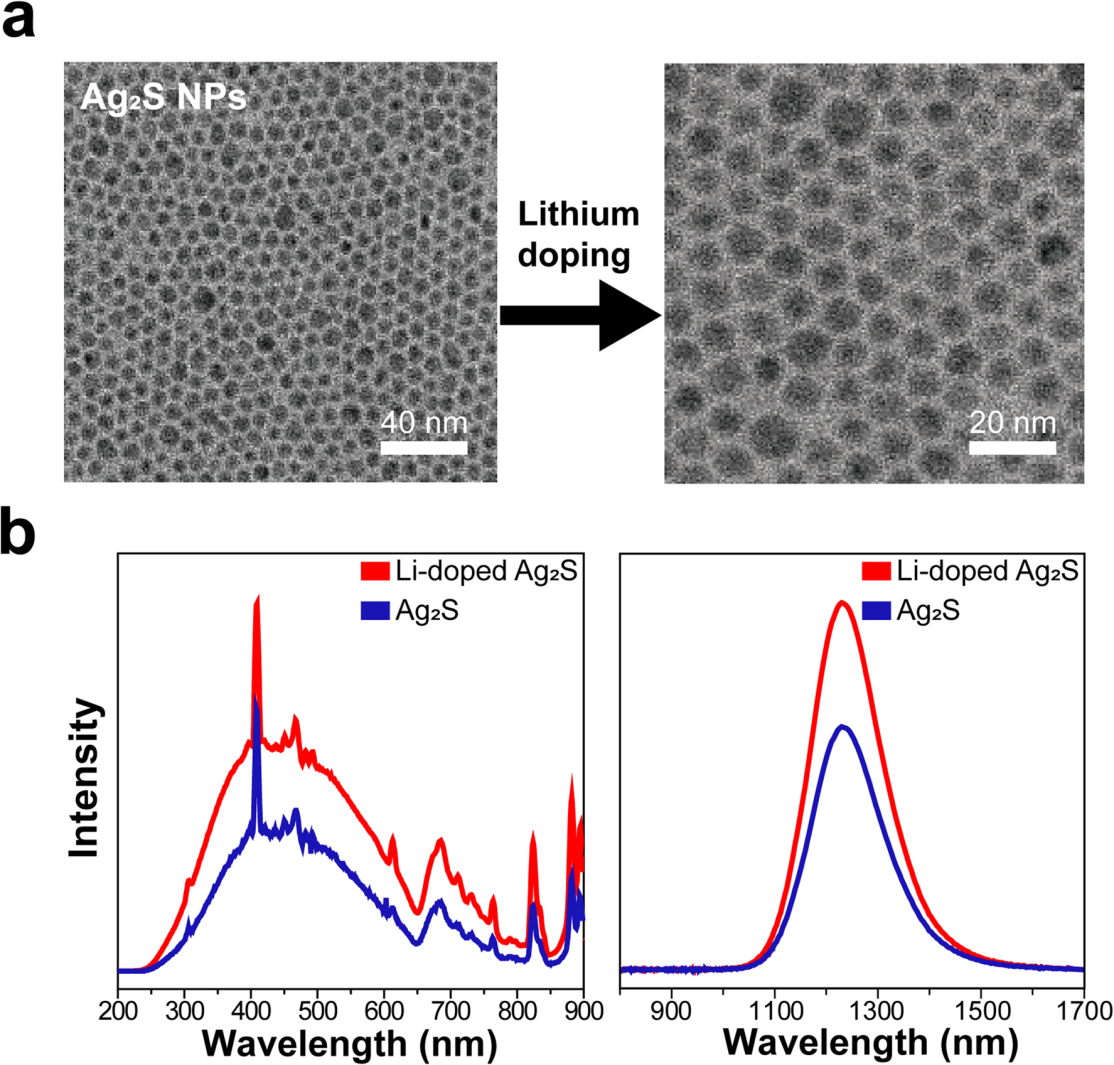
Synthesis of Ag_2_S NPs and Li-doped Ag_2_S NPs. (**a**) Representative TEM images of Ag_2_S NPs (left) and Li-doped Ag_2_S NPs (right) with a 10 nm size. (**b**) PL excitation spectra (left) and emission spectra (right) under excitation from an 850 nm light source.

The structure and morphology of Ag_2_S NPs and Li-doped Ag_2_S NPs were analyzed using a transmission electron microscopy (TEM). Ag_2_S NPs TEM images verified its monodispersity and narrow particle size distribution (Fig. 2a); thus, it can be inferred that phase separation by nucleation and growth processes during ultrasonic irradiation at 160 ℃ is effective. Similar results were published in a previous study^26^. The peaks in the XRD patterns that displayed varying amounts Li^+^ doped Ag_2_S NPs corresponded to the monoclinic Ag_2_S phase (JCPDS No. 014-0072) (Supplementary Fig. S1), and its TEM images displayed monodispersed spherical NPs. Li^+^ concentration did not significantly affect particle morphology, size, and fundamental properties such as the FT-IR spectra and the NPs charges after and before Li^+^ doping except for some differences in the hydrodynamic diameter (Fig. 2a, Supplementary Figs. S2, S4, S5, S6). Figure 2b shows Ag_2_S NPs and Li-doped Ag_2_S NPs PL excitation and emission spectra. Within the UV–NIR range, the NPs can be effectively excited in contrast to PbSe and PbS quantum dots. It has been reported that Ag_2_S NPs, at various excitation ranges, emit efficiently, making them promising candidates in research requiring particular absorption properties in various wavelength regions ranging from UV to NIR. The Li-doped Ag_2_S NPs demonstrated a remarkable augmentation of emission intensity of up to two orders in magnitude when compared with undoped Ag_2_S NPs, exhibiting an emission peak at 1,250 nm. It is well known that Li^+^ ions, even at minimal concentrations, play an essential role as co-dopants in increasing the lumious efficiency of phosphors^30^.

### Effect of NIR emission by Ag_2_S and Li-doped Ag_2_S NPs on human skin cells

**Figure 3:**
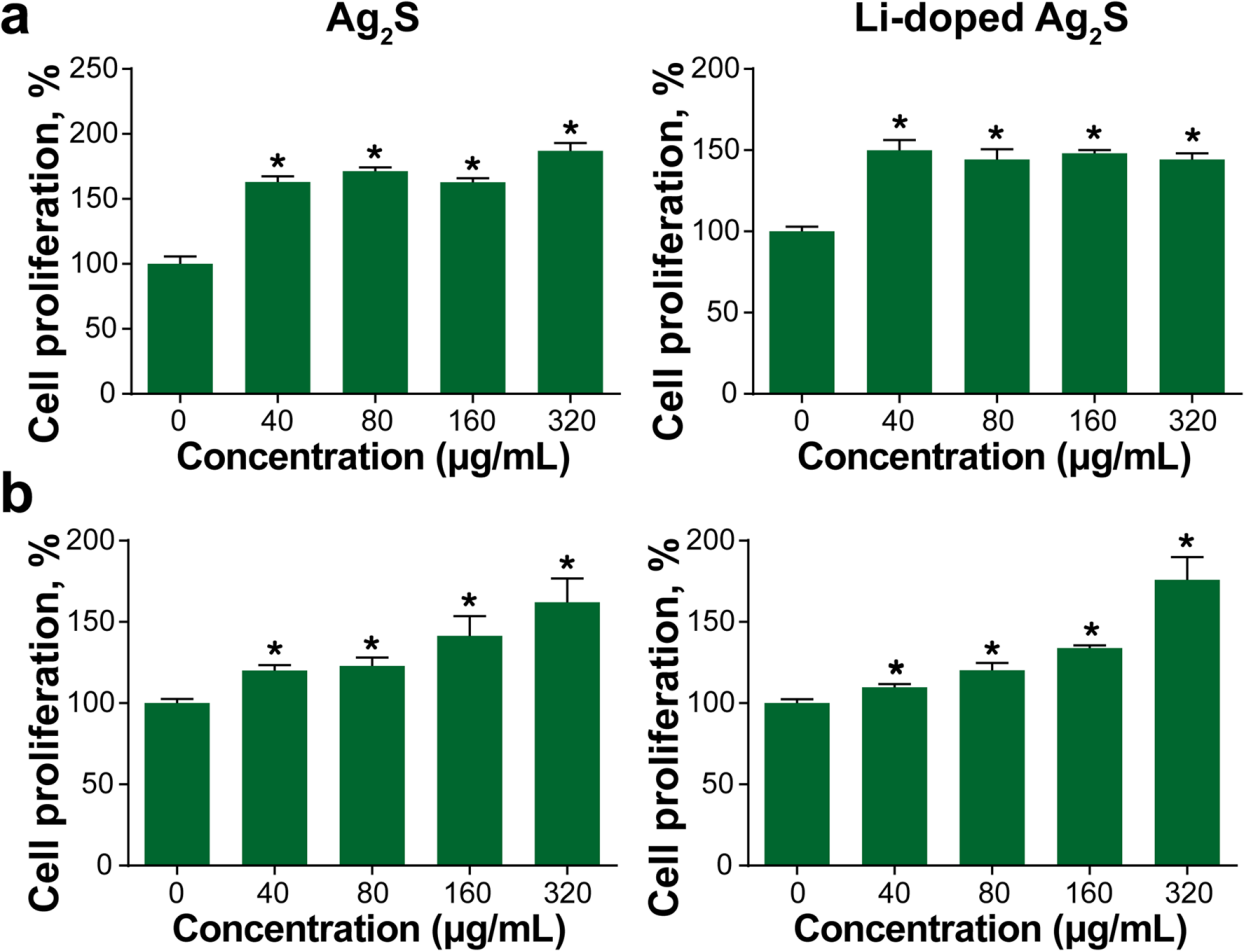
Cell proliferation caused by treatment of Ag_2_S NPs and Li-doped Ag_2_S NPs. (**a**) Cell viability of HaCaT cells after Ag_2_S NP (left) and Li-doped Ag_2_S NP (right) treatment at various concentrations for 24 h. (**b**) Cell viability of HDF cells after Ag_2_S NP (left) and Li-doped Ag_2_S (right) NP treatment at various concentrations for 24 h. The data are expressed as the mean ± SEM. ∗*p* < 0.05 vs 0 (control).

The NIR-emitting properties of Ag_2_S NPs may affect the skin, including wound-healing and anti-wrinkle effects; therefore, we examined their cytotoxicity in HaCaT cells and HDF cells. After 24 h of incubation with NPs, the cell viability was not decreased. Rather, cell proliferation was increased in a dose-dependent manner (Fig. 3a,b). To confirm whether NIR emission was associated with TGF-β signaling and collagen biosynthesis, we exposed HaCaT cells and HDF cells to NIR for 20 min, and the expression of collagen biosynthesis genes was measured by real-time PCR. The expressions of TGF-β1, tissue inhibitor of metalloproteinase 1 (TIMP1), and type 1 collagen (COL1A1) were upregulated by NIR exposure in HaCaT cells and HDF cells when compared with that in cells that were not exposed to light or that had normal light exposure (Supplementary Fig. S3).

**Figure 4:**
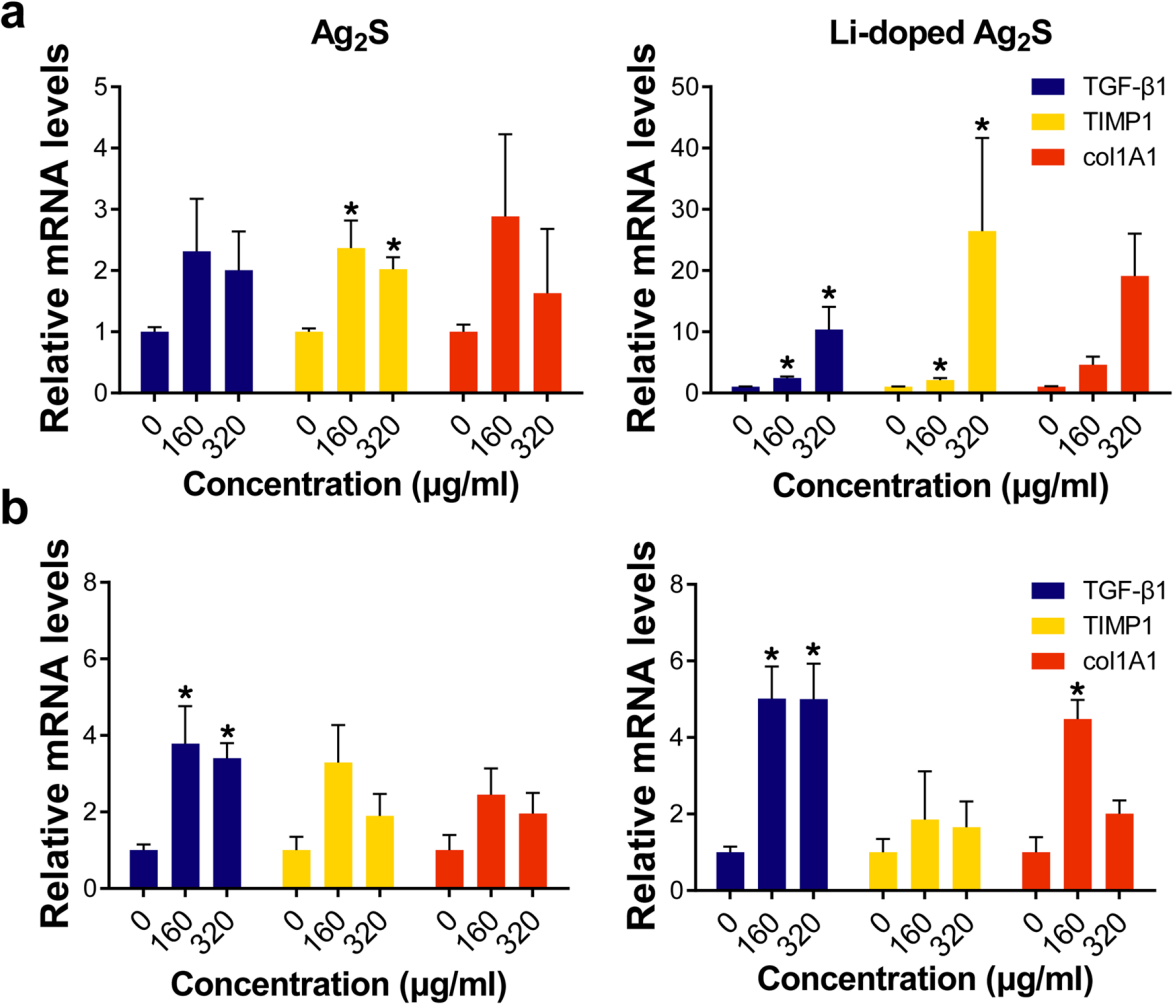
Activation of the TGF-β1 signaling pathway and the upregulation of collagen biosynthesis genes**.** Measurement of collagen biosynthetic genes in HaCaT keratinocytes after (**a**) Ag_2_S (left) and Li-doped Ag_2_S (right) treatment at various concentrations for 24 h. In the same way, (**b**) Ag_2_S (left) and Li-doped Ag_2_S (right) NPs were used to treat HDF cells. The data are expressed as the mean ± SEM. ∗*p* < 0.05 vs 0 (control).

To investigate the role of Ag_2_S NPs and Li-doped Ag_2_S NPs, the expression of genes involved in collagen synthesis was examined in HaCaT cells and HDF cells. HaCaT cells were treated with various concentrations of Ag_2_S NPs and Li-doped Ag_2_S NPs. Only TIMP1 was upregulated when HaCaT cells were treated with Ag_2_S NPs, but in the presence of Li-doped Ag_2_S NPs, there was a dramatic upregulation of the expression of TGF-β1, TIMP1, and COL1A1 (Fig. 4a). In HDF cells, only TGF-β1 was upregulated when cells were treated with Ag_2_S NPs, but both TGF-β1 and COL1A1 were upregulated when cells were treated with Li-doped Ag_2_S NPs (Fig. 4b).

**Figure 5:**
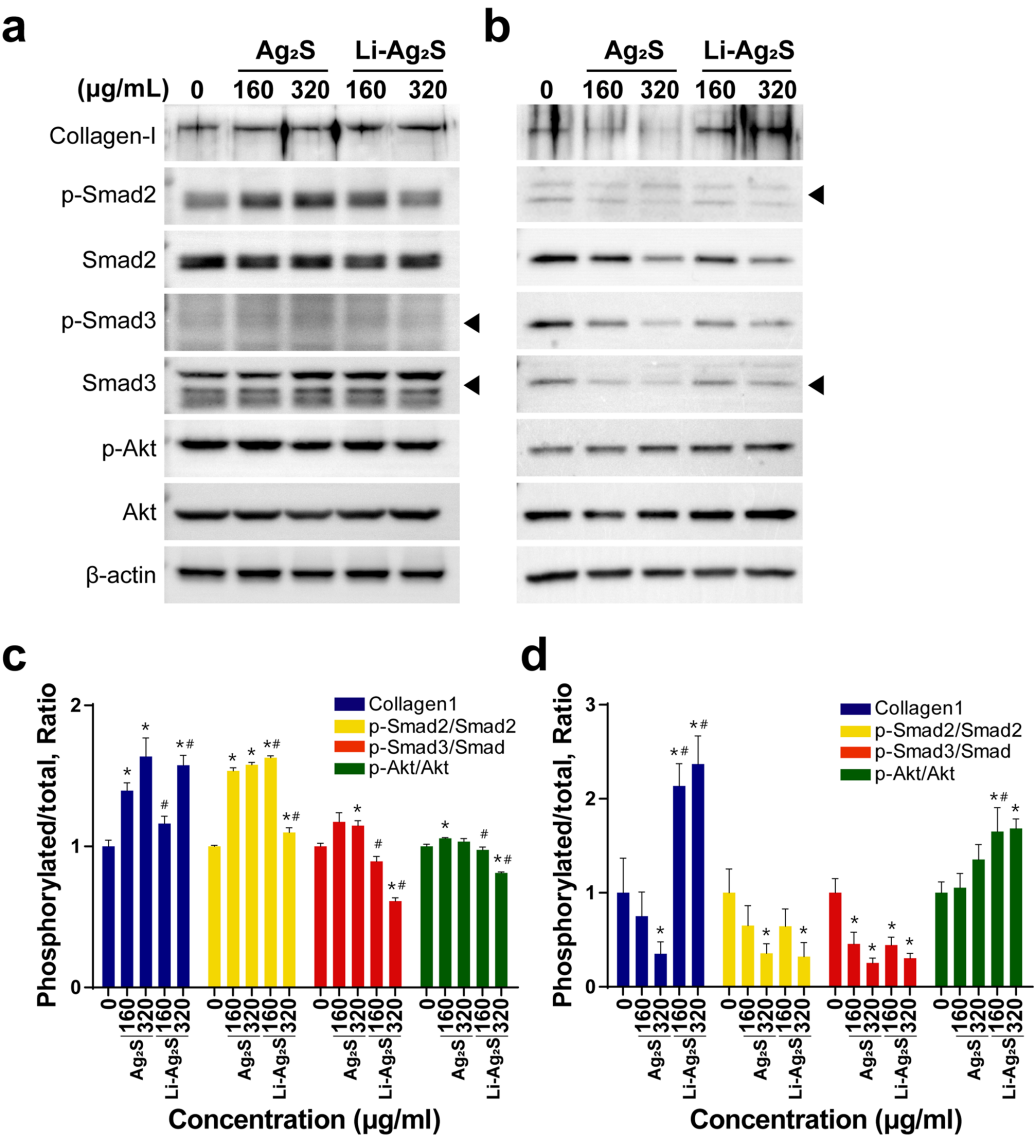
Collagen synthesis resulting from the activation of the TGF-β1 signaling pathway. (**a**) HaCaT cells were treated with either Ag_2_S or Li-doped Ag_2_S NPs at various concentrations for 24 h. (**b**) HDF cells were treated with either Ag_2_S or Li-doped Ag_2_S NPs at various concentrations for 24 h. The levels of intracellular collagen-I, p-Smad2, Smad2, p-Smad3, Smad3, p-Akt and Akt proteins were measured by immunoblot analysis and quantified by Image in (**c**) HaCaT cells and (**d**) HDF cells*.* β-Actin was used as the control. The data are expressed as the mean ± SEM. *p < 0.05 vs. 0 μg/ml control. ^#^p < 0.05 vs. the same treatment with Ag_2_S NPs.

Then, collagen synthesis in HaCaT cells was analyzed by immunoblot analysis to identify the proteins involved in the TGF-β signaling pathway that were upregulated by Ag_2_S NPs or Li-doped Ag_2_S NPs. TGF-β1 binds to the receptors tβRpI and tβRpII, which are located on the surface of the plasma membrane and phosphorylates downstream transcription factors that regulate the expression of target genes such as TGF-β1, TIMP1, and COL1A1. In addition, the Akt signaling pathway, independently of the TGF-β signaling pathway, activates collagen synthesis. When HaCaT cells were treated with Ag_2_S NPs or Li-doped Ag_2_S NPs, the phosphorylation of Smad2 was increased by both types of NPs. In contrast, phosphorylation of Smad3 was slightly increased by Ag_2_S but decreased by Li-doped Ag_2_S (Fig. 5a,c). Additionally, the Akt pathway was determined by immunoblot analysis. Although Akt phosphorylation was not altered by Ag_2_S or Li-doped Ag_2_S NPs, collagen production was increased in the immunoblot analysis (Fig. 5a,c). Fibroblasts are another skin cell type associated with collagen production. Therefore, we examined the effects of NPs on HDF cells. We found that the phosphorylation of Smad2 and Smad3 was decreased by Ag_2_S or Li-doped Ag_2_S NPs. In contrast, the phosphorylation of Akt was increased by Ag_2_S or Li-doped Ag_2_S NPs, and the degree of Akt phosphorylation was higher during Li-doped Ag_2_S NPs treatment (Fig. 5b,d). As a result, collagen synthesis was activated only by Li-doped Ag_2_S NPs. These results suggest that both types of Ag_2_S NPs activate the TGF-β signaling pathway in HaCaT cells and HDF cells and activate collagen synthesis in skin.

### Functional validation of RNA-seq results

**Figure 6:**
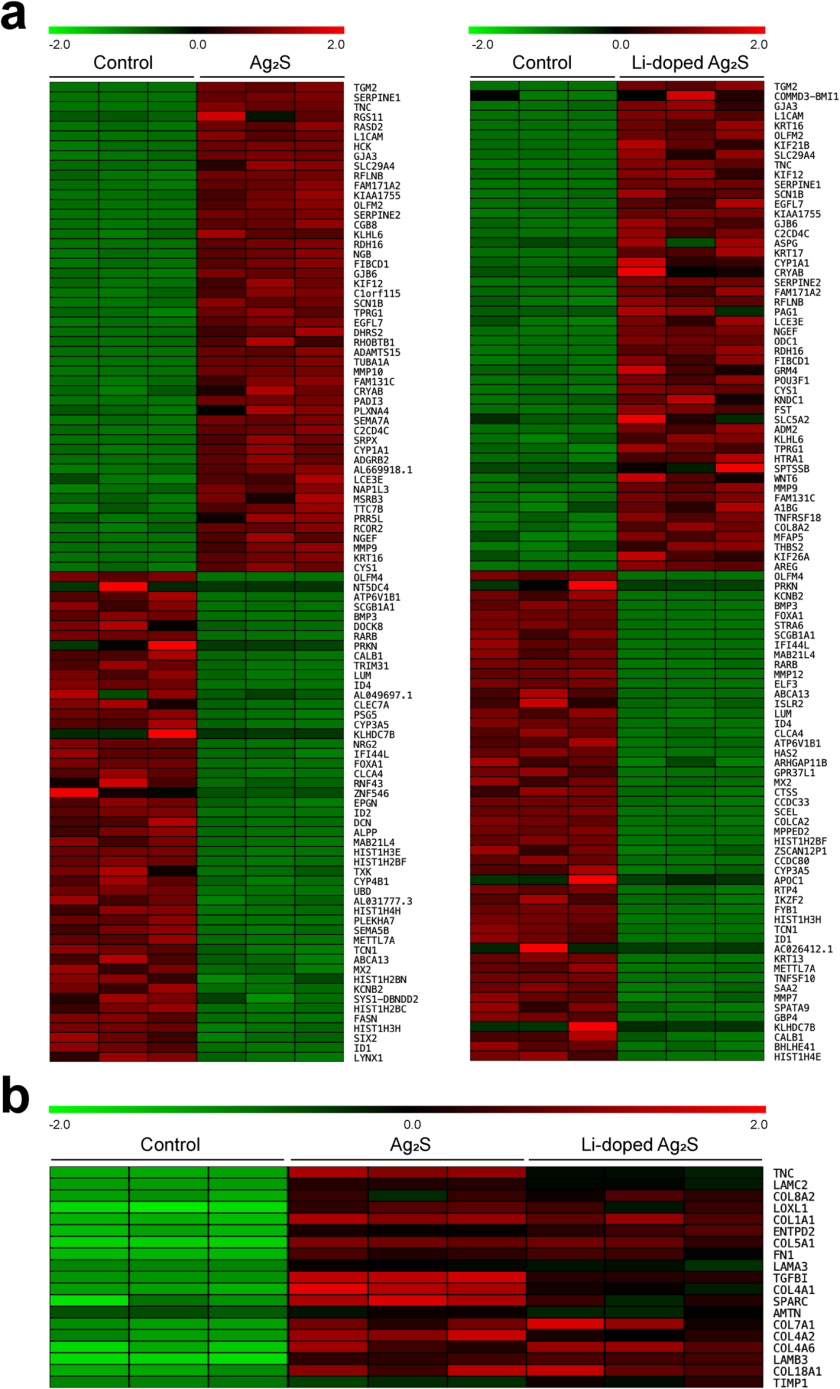
RNA sequencing analysis following treatment of Ag_2_S and/or Li-doped Ag_2_S NPs. (**a**) Heatmap of top 100 genes differentially up-regulated in Ag_2_S- (left) and Li-doped Ag_2_S- (right) treated HaCaT cells. (**b**) Heatmap of 19 genes related extracellular matrix part of cellular component. The gene expression profiling with heatmap showed only the concentration of NPs (320 μg/mL).

To identify various genes that may be involved in the development of dermal fibrosis mediated by NPs in human skin, we performed high-throughput RNA sequencing (RNA-seq) using in vitro HaCaT cells treated with Ag_2_S or Li-doped Ag_2_S NPs at two concentrations (160 and 320 μg/mL) for 24 h. Gene expression profiling identified several novel transcripts in human keratinocyte cells that were significantly up- and down-regulated including the selected targer genes (TGF-β1, TIMP1 and COL1A1), and others (Fig. 6a; Supplementary Tables S1, S2). In a previous study, Gliga et al.^31^ used RNA-seq to measure the effect of Ag-based NPs in human bronchial epithelial cells, and reported that Ag-based NPs are pro-fibrotic and induce epithelial-mesenchymal transition and cell tranformation. We also found a group of 19 genes that make up the extracellular matrix component through gene expression profiling, all of which showed increased expression (Fig. 6b; Supplementary Table S3). However, unlike the real-time PCR results, no dramatic increase depending on NPs concentration was found (data not shown). This is thought to be a discrepancy arising from the sensitivity of the method for measuring gene expression. Among the differentially-regulated genes, Transglutaminase 2 (TGM2) was the most highly up-regulated (log_2_ ratio = 6.93 and 6.33, in Ag_2_S and Li-doped Ag_2_S treated HaCaT cells). TGM2 knockout mice developed significantly reduced pulmonary fibrosis compared with wild-type mice, and overexpression of TGM2 led to increased fibronectin deposition in vitro^32^. The various genes that have been altered by NPs will provide important targets for mechanisms and diseases associated with fibrosis of the skin through future studies. The top 100 most significantly differentially expressed genes regulated by NPs is shown in Supplementary data (Tables S1, S2).

## Discussion

The successful simple preparation of Ag_2_S NPs and Li-doped Ag_2_S NPs can aid the development of NIR therapeutics that can trigger human keratinocytes and collagen to proliferate in the skin microenvironment. Upon ultrasonic radiation, Ag_2_S NPs and Li-doped Ag_2_S NPs enhanced absorption and emission in the NIR region via a one-step process. Further study of the electronic structure of Ag_2_S doped with Li^+^ ion using the first-principles calculations indicated that Li-doped Ag_2_S NPs exhibited increased signalling of NIR photoluminescence and absorption and upregulated collagen expression in skin keratinocytes. Using next-generation sequencing analysis, the increase in expression of various genes constituting the extracellular matrix was observed, and these results are expected to contribute to the study of skin fibrosis in the future. Also, NIR radiation may enhance the wound healing process and increase collagen, and elastin contents form the stimulated fibroblasts, despite the poorly understood biologic effects^2,33^. Ag-based NPs have inherent antibacterial and anti-inflammatory traits, due to its single metallic nanoparticles, that can be altered to develop augmented would and burn dressings^34,35^. Ag-based NPs aid in early wound-healing stages in diabetic patients, although leaving minor scars^36^. Considering applications of Ag-based NPs in wound therapy due to their effective and enhanced antibacterial characteristics, their biocompatibility and safety need to be thoroughly analyzed^37^.

**Figure 7:**
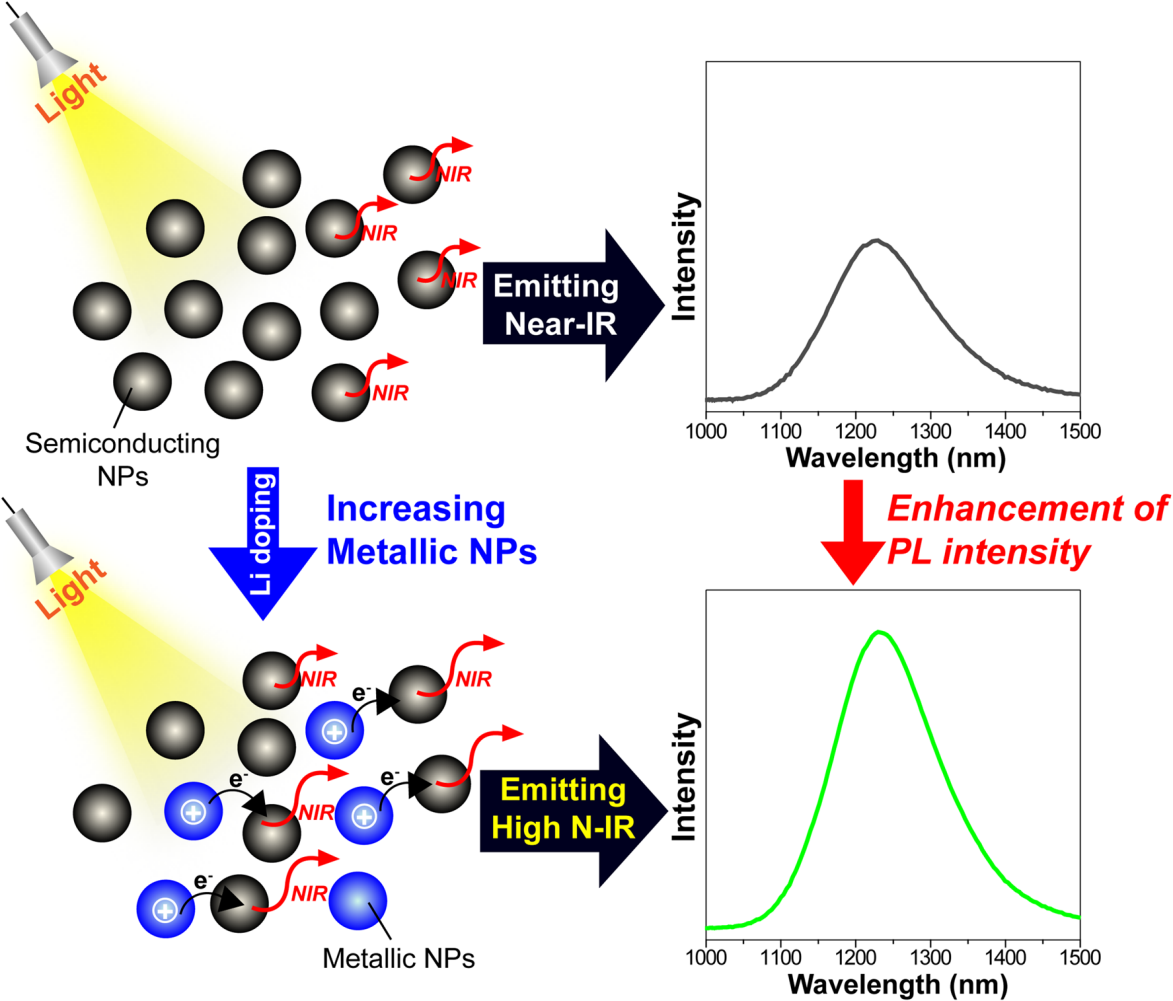
Mechanism of the photoluminescence enhancement of Li-doped Ag_2_S NPs. Schematic diagram showing the mechanism of the photoluminescence enhancement of Ag_2_S NPs by Li^+^ doping. The black and sky-blue balls represent the semiconducting and metallic Ag_2_S NPs, respectively.

In this study, the effects of infrared radiated NPs on collagen expression in human keratinocytes were researched, and results revealed some beneficial properties for skin aging and wound healing. Taken together, we have proposed a photoluminescence enhancement mechanism observed in Fig. 2b shown in Fig. 7. Additional carriers in Ag_2_S NPs interstitially doped with Li^+^ accumulate within the NPs, which further enhances the metallic properties. According to the results of Kang et al.^38^, the electronic structure changes from a semiconducting structure to a metallic band gap structure only in the presence of Li^+^ interstitial doping. Additional electrons begin to migrate to the semiconducting NPs near metallic Ag_2_S NPs as the doping concentration of Li^+^ increases. Accordingly, positively charged metallic NPs can improve the photoluminescence efficiency of the semiconducting NPs. However, this photoluminescence enhancement is only observed in optimum Li^+^ doping conditions. At certain doping conditions, there is less absolute number of semiconducting NPs participating in the photoluminescence process and a decline in their contribution; thus, the total photoluminescence of the sample is reduced. This mechanism of photoluminescence enhancement and quenching has already been proposed in a previous work on Ag-doped CdSe NPs^39^. These properties may be applicable to clinical NIR therapy and the development of anti-wrinkle cosmetics.

## Methods

### Materials

A similar synthesis method and characterization of NPs has been already proposed in a previous study^38^. Silver nitrate [Ag(NO)_3_, 99%], Li_2_CO_3_ (98%), and 1-dodecanethiol (DDT) were purchased from Sigma Aldrich. Chloroform and ethyl acetate were used to disperse and to isolate the NPs. All chemicals were used without further purification.

### Synthesis of Ag_2_S NPs and Li-doped Ag_2_S NPs

The Ag_2_S and Li-doped Ag_2_S NPs were synthesized using a Digital Sonifier 450 ultrasonication from Branson. For the synthesis of Ag_2_S NPs, Ag(NO)_3_ was added to a 20 mL vial containing 10 mL of DDT. The solution was treated by ultrasound irradiation for 10 min in an air atmosphere. The resulting suspension was centrifuged with ethyl acetate several times to remove any by-products and dried in an electric oven at 80 ℃. The Li-doped Ag_2_S NPs were synthesized with the same method upon the addition of the appropriate amount of lithium to the reaction bottle.

### Characterization of Ag_2_S NPs and Li-doped Ag_2_S NPs

The absorption spectra of the solutions containing 0.01 g of Ag_2_S and Li-doped Ag_2_S NPs (in 10 mL of chloroform) were measured using a SolidSpec-3700 UV–Vis–NIR spectrophotometer from Shimadzu. The photoluminescence (PL) spectra were measured by a Fluorolog-3 in TCSPC mode (HORIBA Scientific). All samples were excited by a CW 450 W xenon source, and directed to a single-grating spectrometer. The PL spectra were obtained using an InP/InGaAs detector equipped with an LN cooler. All TEM images were acquired on a JOEL JEM-2100F transmission electron microscope operating at 200 kV. The TEM samples were prepared by drop casting very thin nanoparticle solutions onto a 200 mesh copper grid with a carbon film (Ted Pella). Then X-ray diffraction spectroscopy (XRD) was conducted using a Rigaku D/MAX-220 V X-ray diffractometer coupled with a Cu K-alpha (1.540598 Å) source. The FT-IR was measured by EQUINOX 55 (Bruker). The Zeta potential and DLS were measured by ELS-77 (Otsukael).

### Surface modification of Ag_2_S NPs and Li-doped Ag_2_S NPs with 3-mercaptopropionic acid (MPA)

The Ag_2_S NPs and Li-doped Ag_2_S NPs were subjected to ligand exchange with MPA in its thiolated form, which was created by adjusting the pH value higher than pH 13 by adding 1 M NaOH solution to the 10 mL methanol containing 400 μL MPA. The resulting MPA solution was added drop by drop to the vigorously stirred Ag_2_S NP and Li-doped Ag_2_S NP solutions. After 30 min reaction, the mixed solution was centrifuged and washed with methanol several times. The final product was re-dispersed in distilled water.

### Cell culture and cell viability measurement

HaCaT keratinocyte cells, acquired from the American Type Culture Collection (ATCC, VA, USA), were cultured in high-glucose Dulbecco’s modified Eagle medium (DMEM) supplemented with 10% fetal bovine serum (FBS), 100 U/mL penicillin, and 100 μg/mL streptomycin in 5% CO_2_ under a humidified atmosphere at 37 ℃. Human dermal fibroblast (HDF) cells were obtained from Gibco (CA, USA). The cells were cultured in Medium 106 (Gibco) supplemented with 1 × Low Serum Growth Supplement (LSGS, Gibco) in 5% CO_2_ under a humidified atmosphere at 37 ℃. Cell viability was measure by using an EZ-Cytox Cell viability assay kit (Dail Lab, Korea). HaCaT cells were seeded at 1 × 10^4^ cells/well in 96-well plates and treated with Ag_2_S NPs and Li-doped Ag_2_S NPs at various concentrations for 24 and 48 h. HDF cells were seeded at 3 × 10^3^ cells/well in 96-well plates and treated with Ag_2_S NPs and Li-doped Ag_2_S NPs at various concentrations for 24 h. The WST reagent solution (10 μL) was added to each well of a 96-well microplate that contained 100 μL of cells. The plate was then incubated for 4 h at 37 °C. HDF cells from passage number 10 were used for the study. The absorbance was measured at 450 nm using a microplate reader.

### RNA preparation and real-time PCR

Total RNA was extracted from HaCaT cells using an Easy-spin Total RNA Extraction Kit (Intron Biotechnology, Korea) in accordance with the manufacturer’s instructions. The cDNA was synthesized using an iScript cDNA Synthesis Kit (Bio-Rad, CA, USA), and real-time PCR analysis was conducted with StepOnePlus equipment (Applied Biosystems, CA, USA) using 2 × SYBR Green Master Mix (Takara, Japan). The level of mRNA gene expression was normalized according to the relative expression of β-actin. The primer sequences used in this study are presented in Table 1.

**Table 1:** Primer sequences used in this study.

Target gene	Direction	Sequence (5′–3-)
β-Actin	F R	GAC GTT GAC ATC CGT AAA G CAG TAA CAG TCC GCC T
TGF-β1	F R	CAA TTC CTG GCG ATA CCT CAG GCA CAA CTC CGG TGA CAT CAA
TIMP1	F R	CTT CTG CAA TTC CGA CCT CGT ACG CTG GTA TAA GGT GGT CTG
COL1A1	F R	GAG GGC CAA GAC GAA GAC ATC CAG ATC ACG TCA TCG CAC AAC

### NIR irradiation

The medium was replaced with phosphate-buffered saline (pH 7.2) and using an UIM-250 (Unix, Korea) the cells were exposed to NIR at a distance of 20 cm at room temperature before irradiation. The NIR device emitted NIR spectra between 1,100 and 1,800 nm. The irradiation conditions were 20 J/cm^2^ and 40 J/cm^2^ in this study. It has been reported that under these conditions after immediate exposure to NIR, no temperature rise occurs in the phosphate-buffered saline^40^. As a control, cells were left on a clean bench with natural light during the day time. The cells were maintained in a serum-free medium for 6 h after treatment, and total RNA was extracted using an Easy-spin Total RNA Extraction Kit (Intron Biotechnology, Korea) for mRNA measurement by real-time PCR.

### RNA-sequencing and data analysis

RNA-seq analyses were performed at Theragen Bio Institute (Gyeonggi-do, Korea). The libraries were prepared for 150 bp paired-end sequencing using TruSeq RNA Sample Prep Kit (Illumina, CA, USA). A total of 1 μg of RNA molecules was purified and fragmented, then synthesized as single-stranded cDNAs via random hexamer priming. Using this as a template to synthesize the second strand, a double-stranded cDNA was prepared. cDNA libraries were amplified with PCR after a sequential process of end repair, A-tailing, and adapter ligation. The quality of these cDNA libraries was evaluated with the Agilent 2100 BioAnalyzer (Agilent, CA, USA) and were quantified with the KAPA library quantification kit (Kapa Biosystems, MA, USA) in accordance with the manufacturer’s library quantification protocol. Cluster amplification of denatured templates was followed by paired-end (2 × 150 bp) sequencing using Illumina Novaseq6000 (Illumina, CA, USA). Differential expression analysis was performed by Cuffdiff^41^. With most options set at default values, only multi-read-correction and frag-bias-correct options were applied for better analysis accuracy. DEGs were identified based on the q value threshold of less than 0.05 for correcting errors caused by multiple-testing^42^. The raw datasets of the RNA-seq analysis will be available to the researchers of interest upon request via correspondence.

### Immunoblot analysis

Cells were lysed in RIPA buffer (Biosesang, Korea) containing protease inhibitor. Proteins were quantified using a BCA Protein Assay kit (Thermo Fisher Scientific, MA, USA) and the absorbance was measured at 562 nm using a microplate spectrophotometer (BioTeK Instruments, VT, USA). The protein lysate was used for SDS-PAGE and transferred onto a PVDF membrane. The membrane was blocked with 5% skim milk in TBST (Tris-buffered saline with 0.1% Tween 20) for an hour. Then, it was probed with the following primary antibodies against collagen 1 (dilution 1: 1,000), p-Smad2 (dilution 1: 1,000), Smad2 (dilution 1: 1,000), p-Smad3 (dilution 1: 1,000), Smad3 (dilution 1: 1,000), p-Akt (dilution 1: 1,000), Akt (dilution 1: 1,000), and β-actin (dilution 1: 2,000) overnight at 4 °C. Anti-collagen 1 was purchased from Thermo Fisher Scientific. Anti-pSmad2, anti-Smad2, anti-pSmad3, anti-Smad3, anti-pAkt, and anti-Akt were purchased from Cell Signaling Technology (MA, USA). Anti-β-actin was purchased from Millipore (MA, USA). Then, the blots were incubated with horseradish peroxidase (HRP)-linked goat anti-rabbit (or mouse) secondary IgG (Bio-Rad Laboratories, CA, USA) at room temperature for an hour. The signals were detected by SuperSignal West Pico Chemiluminescent Substrate (Thermo Fisher Scientific). A chemiluminescence image analyzer (Vilber Lourmat, France) was used for the detection of the signals.

## Supplementary information

Supplementary information.
