# Supplementary material for: Near-infrared-emitting nanoparticles activate collagen synthesis via TGFβ signaling
Source: Sci Rep. 2020 Aug 6;10:13309. doi: 10.1038/s41598-020-70415-1 (PMC7410846; doi:10.1038/s41598-020-70415-1)
Supplement: Supplementary file 1 — Supplementary information. [file 41598_2020_70415_MOESM1_ESM.pdf]

Near-infrared-emitting nanoparticles activate collagen synthesis via TGFβ signaling

Myung Hyun Kang[1], Han Young Yu[2], Goon-Tae Kim[2], Ji Eun Lim[1], Seunghun Jang[3], Tae-Sik Park[2,*] & Joung Kyu Park[1,*]

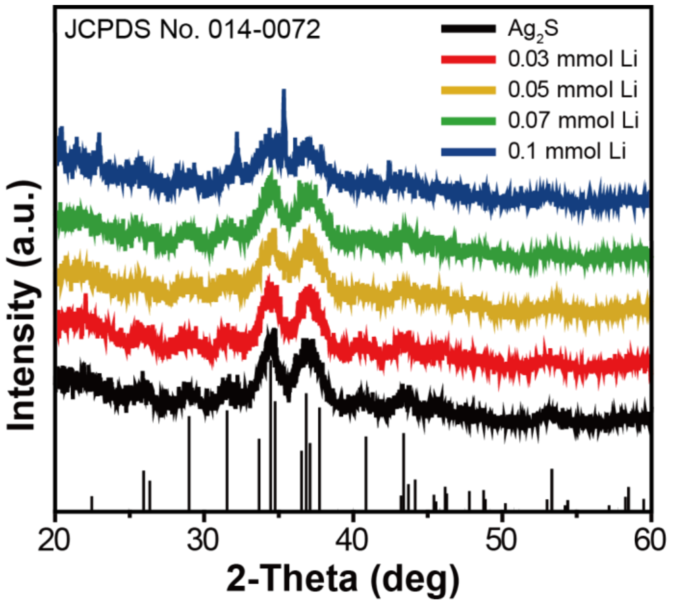

Supplementary Figure 1. XRD Patterns of Ag_2S NPs in terms of different amounts of Li^+.

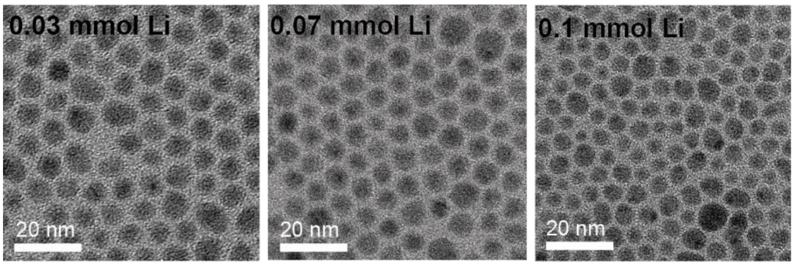

Supplementary Figure 2. TEM images of Ag_2S NPs for different amounts of Li^+.

left: 0.03 mmol Li-doped, middle: 0.07 mmol Li-doped, right: 0.1 mmol Li-doped Ag_2S NPs.

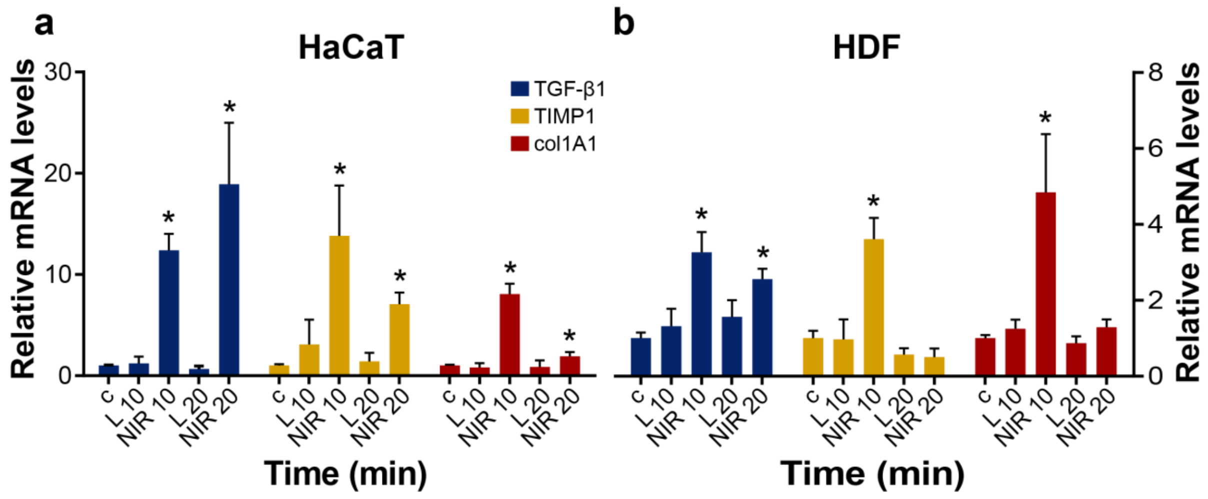

Supplementary Figure 3. Upregulation of TGFβ1, TIMP1 and col1A1 by NIR irradiation. L: natural light exposure, NIR: near-infrared exposure for 10 or 20 min. **a** HaCaT cells were exposed to NIR. **b** HDF cells were exposed to NIR. The data were expressed as mean ± SEM. $*p < 0.05$ vs C (control).

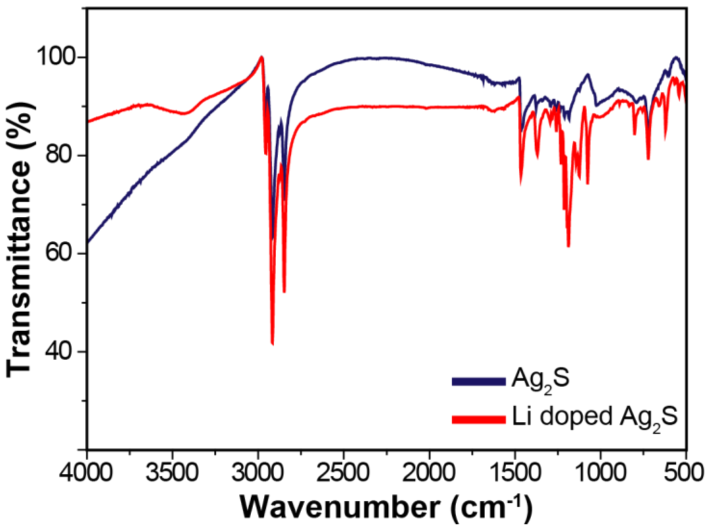

Supplementary Figure 4. FT-IR Spectrum of Ag_2S and Li-doped Ag_2S

	Zeta potential (mV)	PDI	Z-Average (d.nm)
**Ag_2S**	31.73	0.560	25.67
**MPA coated Ag_2S**	-32.21	0.139	59.2
**Li doped Ag_2S**	26.38	0.379	53.08
**MPA coated Li-Ag_2S**	-30.17	0.185	63.36

Supplementary Figure 5. Zeta potential and DLS of Ag_2S, Li-Ag_2S, MPA-coated Ag_2S, and MPA-coated Li-Ag_2S NPs.

a)

			Size (d.nm):	% Number:	St Dev (d.nm):
Z-Average (d.nm):	25.67	Peak 1:	2.360	100.0	0.6487
PdI:	0.560	Peak 2:	0.000	0.0	0.000
Intercept:	0.151	Peak 3:	0.000	0.0	0.000

Result quality : Refer to quality report

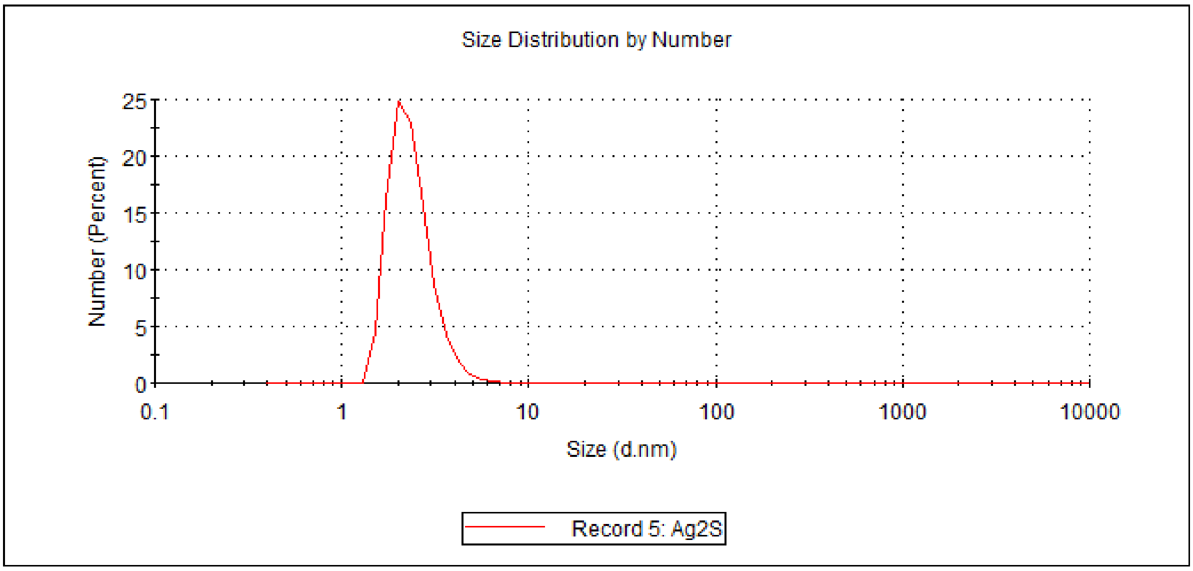

b)

			Size (d.nm):	% Number:	St Dev (d.nm):
Z-Average (d.nm):	59.20	Peak 1:	40.18	100.0	10.64
PdI:	0.139	Peak 2:	0.000	0.0	0.000
Intercept:	0.954	Peak 3:	0.000	0.0	0.000

Result quality : Refer to quality report

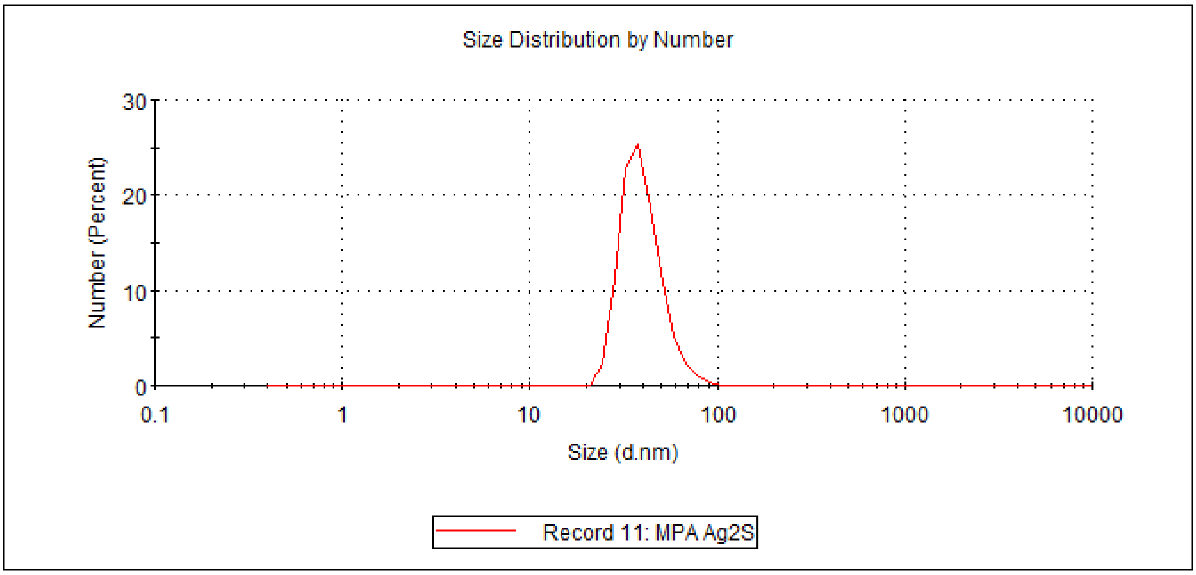

c)

			Size (d.nm):	% Number:	St Dev (d.nm):
Z-Average (d.nm):	53.08	Peak 1:	14.29	100.0	4.113
PdI:	0.379	Peak 2:	0.000	0.0	0.000
Intercept:	0.191	Peak 3:	0.000	0.0	0.000

Result quality : Refer to quality report

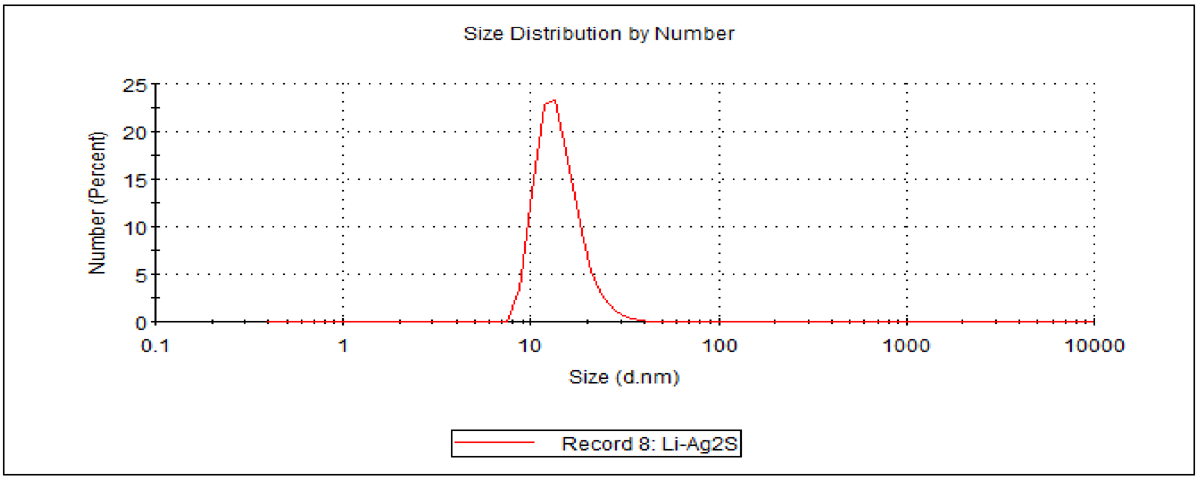

d)

			Size (d.nm):	% Number:	St Dev (d.nm):
Z-Average (d.nm):	63.36	Peak 1:	40.72	100.0	10.96
PdI:	0.185	Peak 2:	0.000	0.0	0.000
Intercept:	0.952	Peak 3:	0.000	0.0	0.000

Result quality : Refer to quality report

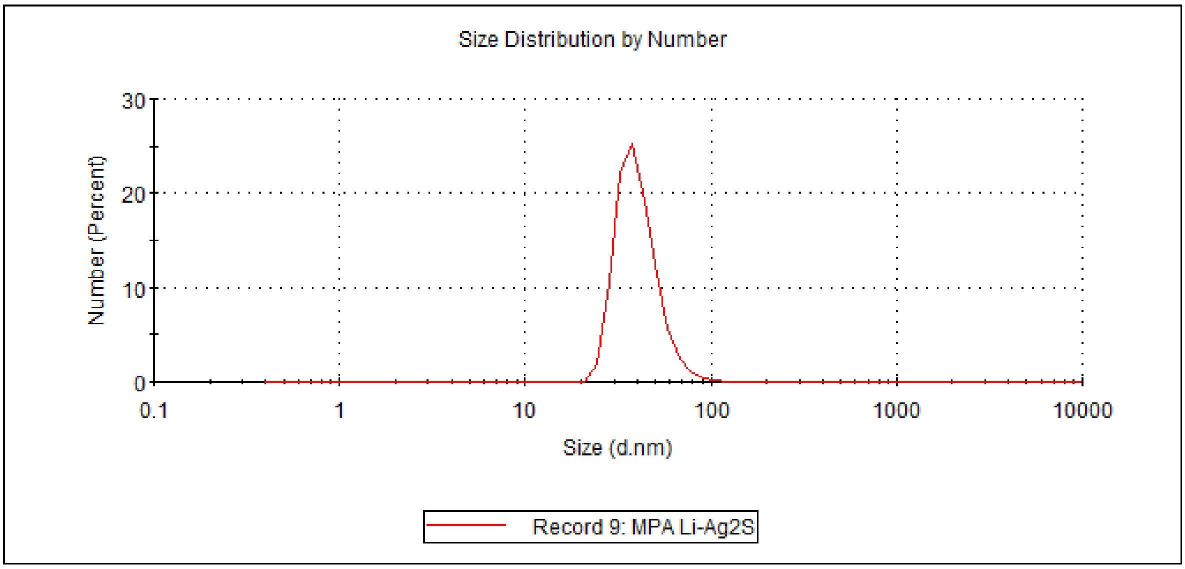

Supplementary Figure 6. Dynamic Light Scattering(DLS) spectra of Ag_2S, Li-doped Ag_2S, MPA-coated Ag_2S and MPA-coated Li-Ag_2S. a) Ag_2S, b) MPA-coated Ag_2S, c) Li-doped Ag_2S, d) MPA-coated Li-Ag_2S

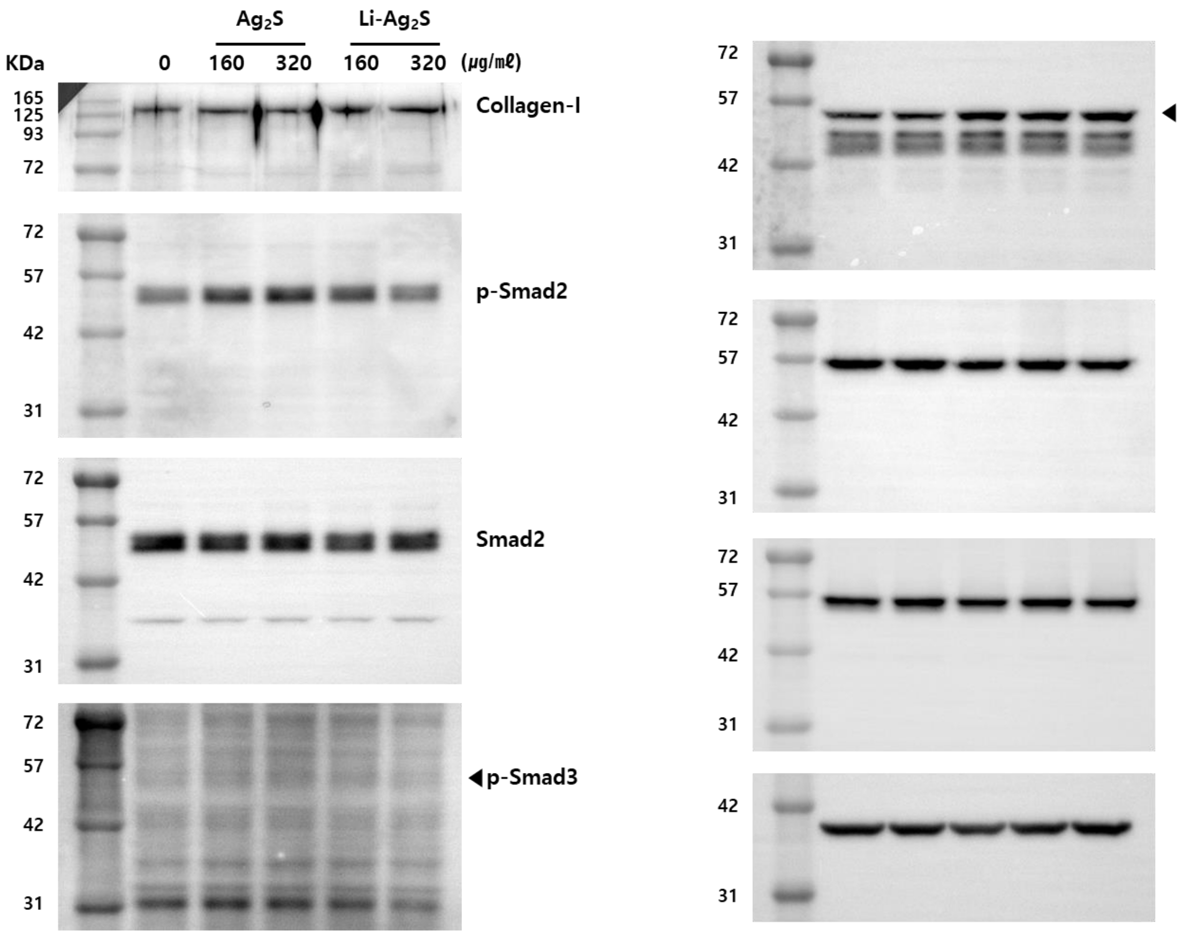

Supplementary Figure 7. Uncropped images of Figure 5a

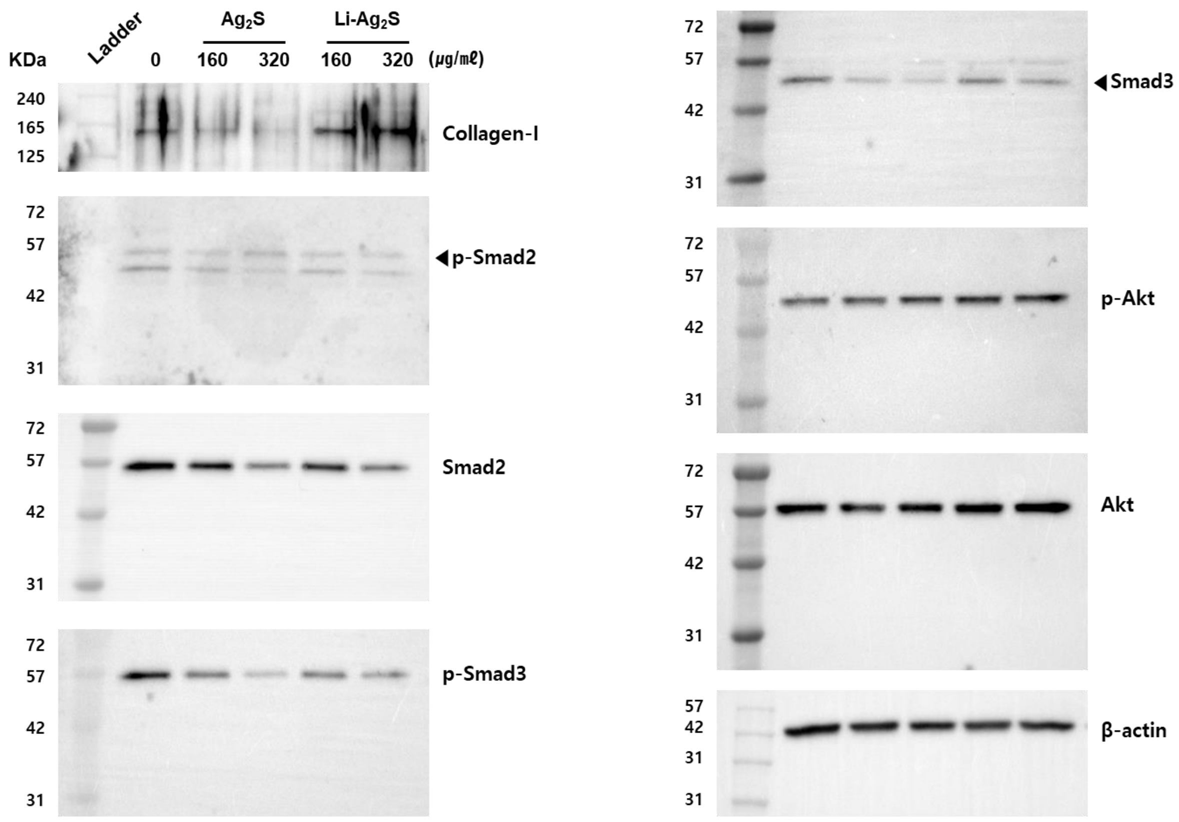

Supplementary Figure 8. Uncropped images of Figure 5b

Supplementary Table 1. List of top 100 genes differentially up and down-regulated in Ag_2S treated keratinocytes

#	Gene Symbol	Description	Log2 ratio	p-value
Up-regulated genes				
1	TGM2	transglutaminase 2	6.93	0.00005
2	SERPINE1	serpin family E member 1	5.68	0.00005
3	TNC	tenascin C	5.46	0.00005
4	L1CAM	L1 cell adhesion molecule	4.97	0.00005
5	RFLNB	refilin B	4.47	0.00005
6	FAM171A2	family with sequence similarity 171 member A2	4.40	0.00005
7	KIAA1755	KIAA1755	4.32	0.00005
8	OLFM2	olfactomedin 2	4.19	0.00005
9	SERPINE2	serpin family E member 2	4.19	0.00005
10	KLHL6	kelch like family member 6	4.10	0.00005
11	RDH16	retinol dehydrogenase 16	4.08	0.00005
12	NGB	neuroglobin	4.03	0.00005
13	FIBCD1	fibrinogen C domain containing 1	3.99	0.00005
14	GJB6	gap junction protein beta 6	3.95	0.00005
15	C1orf115	chromosome 1 open reading frame 115	3.89	0.00005
16	SCN1B	sodium voltage-gated channel beta subunit 1	3.89	0.00005
17	TPRG1	tumor protein p63 regulated 1	3.87	0.00005
18	EGFL7	EGF like domain multiple 7	3.86	0.00005
19	DHRS2	dehydrogenase/reductase 2	3.82	0.00005
20	RHOBTB1	Rho related BTB domain containing 1	3.81	0.00005
21	ADAMTS15	ADAM metallopeptidase with thrombospondin type 1 motif 15	3.76	0.00005
22	TUBA1A	tubulin alpha 1a	3.75	0.00005
23	MMP10	matrix metallopeptidase 10	3.74	0.00005
24	FAM131C	family with sequence similarity 131 member C	3.73	0.00005
25	CRYAB	crystallin alpha B	3.71	0.00005
26	PADI3	peptidyl arginine deiminase 3	3.68	0.00005
27	SEMA7A	semaphorin 7A (John Milton Hagen blood group)	3.65	0.00005
28	C2CD4C	C2 calcium dependent domain containing 4C	3.62	0.00005
29	CYP1A1	cytochrome P450 family 1 subfamily A member 1	3.61	0.00005
30	ADGRB2	adhesion G protein-coupled receptor B2	3.60	0.00005
31	MSRB3	methionine sulfoxide reductase B3	3.49	0.00005
32	TTC7B	tetratricopeptide repeat domain 7B	3.47	0.00005
33	MMP9	matrix metallopeptidase 9	3.46	0.00005
34	NGEF	neuronal guanine nucleotide exchange factor	3.46	0.00005
35	PRR5L	proline rich 5 like	3.46	0.00005
36	RCOR2	REST corepressor 2	3.46	0.00005
37	KRT16	keratin 16	3.40	0.00005
38	CYS1	cystin 1	3.39	0.00005
39	LAMC2	laminin subunit gamma 2	3.37	0.00005
40	DACT1	dishevelled binding antagonist of beta catenin 1	3.35	0.00005
41	SH3GL3	SH3 domain containing GRB2 like 3, endophilin A3	3.32	0.00005
42	HS3ST2	heparan sulfate-glucosamine 3-sulfotransferase 2	3.29	0.00005
43	IL1A	interleukin 1 alpha	3.27	0.00005
44	GJB2	gap junction protein beta 2	3.23	0.00005
45	COL8A2	collagen type VIII alpha 2 chain	3.21	0.00005
46	KRT17	keratin 17	3.19	0.00005
47	CYP2R1	cytochrome P450 family 2 subfamily R member 1	3.18	0.00005
48	HCN2	hyperpolarization activated cyclic nucleotide gated potassium and sodium channel 2	3.17	0.00005
49	KNDC1	kinase non-catalytic C-lobe domain containing 1	3.16	0.00005
50	ODC1	ornithine decarboxylase 1	3.15	0.00005
Down-regulated genes				
51	OLFM4	Olfactomedin 4	-7.75	0.00005
52	ATP6V1B1	Atpase H+ transporting V1 subunit B1	-5.13	0.00005
53	SCGB1A1	Secretoglobin family 1A member 1	-5.02	0.00005
54	BMP3	Bone morphogenetic protein 3	-4.91	0.00005
55	DOCK8	Dedicator of cytokinesis 8	-4.64	0.00005
56	PRKN	Parkin RBR E3 ubiquitin protein ligase	-4.62	0.00005
57	CALB1	Calbindin 1	-4.58	0.00005
58	TRIM31	Tripartite motif containing 31	-4.30	0.00005
59	LUM	Lumican	-4.06	0.00005
60	ID4	Inhibitor of DNA binding 4, HLH protein	-4.05	0.00005
61	CLEC7A	C-type lectin domain containing 7A	-3.98	0.00005

62	PSG5	Pregnancy specific beta-1-glycoprotein 5	-3.85	0.00005
63	CYP3A5	Cytochrome P450 family 3 subfamily A member 5	-3.82	0.00005
64	KLHDC7B	Kelch domain containing 7B	-3.74	0.00005
65	NRG2	Neuregulin 2	-3.61	0.00005
66	IFI44L	Interferon induced protein 44 like	-3.55	0.00005
67	FOXA1	Forkhead box A1	-3.54	0.00005
68	CLCA4	Chloride channel accessory 4	-3.52	0.00005
69	ID2	Inhibitor of DNA binding 2	-3.34	0.00005
70	DCN	Decorin	-3.31	0.00005
71	ALPP	Alkaline phosphatase, placental	-3.30	0.00005
72	MAB21L4	Mab-21 like 4	-3.28	0.00005
73	HIST1H3E	Histone cluster 1 H3 family member e	-3.11	0.00005
74	HIST1H2BF	Histone cluster 1 H2B family member f	-3.06	0.00005
75	CYP4B1	Cytochrome P450 family 4 subfamily B member 1	-2.96	0.00005
76	UBD	Ubiquitin D	-2.94	0.00005
77	AL031777.3	Novel protein	-2.84	0.00005
78	HIST1H4H	Histone cluster 1 H4 family member h	-2.78	0.00005
79	PLEKHA7	Pleckstrin homology domain containing A7	-2.74	0.00005
80	SEMA5B	Semaphorin 5B	-2.74	0.00005
81	METTL7A	Methyltransferase like 7A	-2.68	0.00005
82	TCN1	Transcobalamin 1	-2.67	0.00005
83	ABCA13	ATP binding cassette subfamily A member 13	-2.63	0.00005
84	MX2	MX dynamin like gtpase 2	-2.61	0.00005
85	HIST1H2BN	Histone cluster 1 H2B family member n	-2.53	0.00005
86	KCNB2	Potassium voltage-gated channel subfamily B member 2	-2.52	0.00005
87	HIST1H2BC	Histone cluster 1 H2B family member c	-2.49	0.00005
88	FASN	Fatty acid synthase	-2.48	0.00005
89	HIST1H3H	Histone cluster 1 H3 family member h	-2.46	0.00005
90	ID1	Inhibitor of DNA binding 1, HLH protein	-2.40	0.00005
91	HIST1H2BJ	Histone cluster 1 H2B family member j	-2.37	0.00005
92	ELF3	E74 like ETS transcription factor 3	-2.35	0.00005
93	MPPED2	Metallophosphoesterase domain containing 2	-2.32	0.00005
94	ISPD	Isoprenoid synthase domain containing	-2.30	0.00005
95	CCDC80	Coiled-coil domain containing 80	-2.28	0.00005
96	MX1	MX dynamin like gtpase 1	-2.24	0.00005
97	HERC6	HECT and RLD domain containing E3 ubiquitin protein ligase family member 6	-2.19	0.00005
98	RTP4	Receptor transporter protein 4	-2.19	0.00005
99	COLCA2	Colorectal cancer associated 2	-2.14	0.00005
100	HIST1H3G	Histone cluster 1 H3 family member g	-2.10	0.00005

Supplementary Table 2. List of top 100 genes differentially up-regulated in Li-doped Ag_2S treated keratinocytes

#	Gene Symbol	Description	Log2 ratio	p-value
Up-regulated genes				
1	TGM2	Transglutaminase 2	6.33	0.00005
2	L1CAM	L1 cell adhesion molecule	5.29	0.00005
3	KRT16	Keratin 16	5.05	0.00005
4	OLFM2	Olfactomedin 2	4.90	0.00005
5	KIF21B	Kinesin family member 21B	4.59	0.00005
6	TNC	Tenascin C	4.53	0.00005
7	SERPINE1	Serpin family E member 1	4.46	0.00005
8	SCN1B	Sodium voltage-gated channel beta subunit 1	4.28	0.00005
9	EGFL7	EGF like domain multiple 7	4.20	0.00005
10	KIAA1755	Kiaa1755	4.17	0.00005
11	GJB6	Gap junction protein beta 6	4.16	0.00005
12	C2CD4C	C2 calcium dependent domain containing 4C	4.14	0.00005
13	ASPG	Asparaginase	4.10	0.00005
14	KRT17	Keratin 17	4.10	0.00005
15	CYP1A1	Cytochrome P450 family 1 subfamily A member 1	4.09	0.00005
16	CRYAB	Crystallin alpha B	4.07	0.00005
17	SERPINE2	Serpin family E member 2	4.07	0.00005
18	FAM171A2	Family with sequence similarity 171 member A2	4.04	0.00005
19	RFLNB	Refilin B	3.98	0.00005
20	NGEF	Neuronal guanine nucleotide exchange factor	3.88	0.00005
21	ODC1	Ornithine decarboxylase 1	3.87	0.00005
22	RDH16	Retinol dehydrogenase 16	3.85	0.00005
23	FIBCD1	Fibrinogen C domain containing 1	3.80	0.00005
24	GRM4	Glutamate metabotropic receptor 4	3.75	0.00005
25	CYS1	Cystin 1	3.69	0.00005
26	KNDC1	Kinase non-catalytic C-lobe domain containing 1	3.67	0.00005
27	FST	Follistatin	3.67	0.00005
28	ADM2	Adrenomedullin 2	3.66	0.00005
29	KLHL6	Kelch like family member 6	3.62	0.00005
30	TPRG1	Tumor protein p63 regulated 1	3.61	0.00005
31	HTRA1	Htra serine peptidase 1	3.59	0.00005
32	SPTSSB	Serine palmitoyltransferase small subunit B	3.54	0.00005
33	WNT6	Wnt family member 6	3.52	0.00005
34	MMP9	Matrix metallopeptidase 9	3.52	0.00005
35	FAM131C	Family with sequence similarity 131 member C	3.50	0.00005
36	COL8A2	Collagen type VIII alpha 2 chain	3.47	0.00005
37	MFAP5	Microfibril associated protein 5	3.46	0.00005
38	KIF26A	Kinesin family member 26A	3.40	0.00005
39	AREG	Amphiregulin	3.38	0.00005
40	MMP10	Matrix metallopeptidase 10	3.37	0.00005
41	ZNF467	Zinc finger protein 467	3.37	0.00005
42	COL5A3	Collagen type V alpha 3 chain	3.35	0.00005
43	PRR5L	Proline rich 5 like	3.34	0.00005
44	RCOR2	REST corepressor 2	3.34	0.00005
45	NEFL	Neurofilament light	3.34	0.00005
46	TUBA1A	Tubulin alpha 1a	3.26	0.00005
47	CHAC1	Chac glutathione specific gamma-glutamylcyclotransferase 1	3.25	0.00005
48	ADGRB2	Adhesion G protein-coupled receptor B2	3.24	0.00005
49	TTC7B	Tetratricopeptide repeat domain 7B	3.24	0.00005
50	ACAP1	Arfgap with coiled-coil, ankyrin repeat and PH domains 1	3.24	0.00005
Down-regulated genes				
51	OLFM4	Olfactomedin 4	-8.37	0.00005
52	BMP3	Bone morphogenetic protein 3	-5.80	0.00005
53	FOXA1	Forkhead box A1	-5.75	0.00005
54	STRA6	Stimulated by retinoic acid 6	-5.01	0.00005
55	SCGB1A1	Secretoglobin family 1A member 1	-4.96	0.00005
56	IFI44L	Interferon induced protein 44 like	-4.89	0.00005
57	MAB21L4	Mab-21 like 4	-4.81	0.00005
58	MMP12	Matrix metallopeptidase 12	-4.43	0.00005
59	ELF3	E74 like ETS transcription factor 3	-4.25	0.00005
60	ABCA13	ATP binding cassette subfamily A member 13	-4.10	0.00005
61	ISLR2	Immunoglobulin superfamily containing leucine rich repeat 2	-3.83	0.00005
62	ID4	Inhibitor of DNA binding 4, HLH protein	-3.77	0.00005
63	CLCA4	Chloride channel accessory 4	-3.73	0.00005

64	HAS2	Hyaluronan synthase 2	-3.53	0.00005
65	MX2	MX dynamin like gtpase 2	-3.26	0.00005
66	CTSS	Cathepsin S	-3.24	0.00005
67	CCDC33	Coiled-coil domain containing 33	-3.23	0.00005
68	SCEL	Sciellin	-3.18	0.00005
69	COLCA2	Colorectal cancer associated 2	-3.15	0.00005
70	MPPED2	Metallophosphoesterase domain containing 2	-3.11	0.00005
71	HIST1H2BF	Histone cluster 1 H2B family member f	-3.08	0.00005
72	CCDC80	Coiled-coil domain containing 80	-2.96	0.00005
73	CYP3A5	Cytochrome P450 family 3 subfamily A member 5	-2.87	0.00005
74	RTP4	Receptor transporter protein 4	-2.82	0.00005
75	IKZF2	IKAROS family zinc finger 2	-2.77	0.00005
76	FYB1	FYN binding protein 1	-2.74	0.00005
77	HIST1H3H	Histone cluster 1 H3 family member h	-2.72	0.00005
78	ID1	Inhibitor of DNA binding 1, HLH protein	-2.69	0.00005
79	AC026412.1	Programmed cell death 6 pseudogene	-2.60	0.00005
80	KRT13	Keratin 13	-2.58	0.00005
81	METTL7A	Methyltransferase like 7A	-2.56	0.00005
82	TNFSF10	TNF superfamily member 10	-2.56	0.00005
83	SAA2	Serum amyloid A2	-2.54	0.00005
84	MMP7	Matrix metallopeptidase 7	-2.54	0.00005
85	GBP4	Guanylate binding protein 4	-2.52	0.00005
86	KLHDC7B	Kelch domain containing 7B	-2.50	0.00005
87	CALB1	Calbindin 1	-2.50	0.00005
88	BHLHE41	Basic helix-loop-helix family member e41	-2.47	0.00005
89	GAS2L3	Growth arrest specific 2 like 3	-2.44	0.00005
90	KIF20A	Kinesin family member 20A	-2.40	0.00005
91	GCNT4	Glucosaminyl (N-acetyl) transferase 4, core 2	-2.39	0.00005
92	DHRS3	Dehydrogenase/reductase 3	-2.36	0.00005
93	SGO1	Shugoshin 1	-2.35	0.00005
94	KRT4	Keratin 4	-2.34	0.00005
95	ID2	Inhibitor of DNA binding 2	-2.33	0.00005
96	HIST1H2BC	Histone cluster 1 H2B family member c	-2.33	0.00005
97	MKI67	Marker of proliferation Ki-67	-2.32	0.00005
98	KLK13	Kallikrein related peptidase 13	-2.30	0.00005
99	NRG2	Neuregulin 2	-2.26	0.00005
100	TRIM22	Tripartite motif containing 22	-2.23	0.00005

Supplementary Table 3. List of 19 genes related extracellular matrix part of cellular component

Gene Symbol	Description	log_2 ratio (Fold change > 2)	
		Ag_2S	Li-doped Ag_2S
TNC	Tenascin C	5.46	4.53
LAMC2	Laminin subunit gamma 2	3.37	3.11
COL8A2	Collagen type VIII alpha 2 chain	3.21	3.47
LOXL1	Lysyl oxidase like 1	2.87	2.85
COL1A1	Collagen type I alpha 1 chain	2.81	2.72
ENTPD2	Ectonucleoside triphosphate diphosphohydrolase 2	2.70	3.07
COL5A1	Collagen type V alpha 1 chain	2.69	2.70
FN1	Fibronectin 1	2.48	2.55
LAMA3	Laminin subunit alpha 3	2.45	2.31
TGFBI	Transforming growth factor beta induced	2.37	1.87
COL4A1	Collagen type IV alpha 1 chain	2.04	1.45
SPARC	Secreted protein acidic and cysteine rich	1.77	1.32
AMTN	Amelotin	1.52	1.35
COL7A1	Collagen type VII alpha 1 chain	1.42	1.67
COL4A2	Collagen type IV alpha 2 chain	1.35	1.04
COL4A6	Collagen type IV alpha 6 chain	1.24	1.47
LAMB3	Laminin subunit beta 3	1.10	1.24
COL18A1	Collagen type XVIII alpha 1 chain	1.08	1.18
TIMP1	TIMP metallopeptidase inhibitor 1	1.02	1.62
